# Micro‐QuEChERS Extraction for Broad‐Spectrum LC‐QQQ‐MS Quantification of Nitazene Analogs in Plasma: A Validated Method With Casework Application

**DOI:** 10.1002/dta.70126

**Published:** 2026-07-28

**Authors:** Karla A. O. Souza, Náthaly C. B. F. Santos, Rafael Lanaro, Sara E. Walton, Alex J. Krotulski, Barry K. Logan, Mauricio Yonamine, Jose Luiz Costa

**Affiliations:** ^1^ Centro de Informação e Assistência Toxicológica de Campinas (CIATox‐Campinas) Universidade Estadual de Campinas (UNICAMP) Campinas SP Brazil; ^2^ Faculdade de Ciências Médicas Universidade Estadual de Campinas (UNICAMP) Campinas SP Brazil; ^3^ Center for Forensic Science Research and Education Fredric Rieders Family Foundation Horsham Pennsylvania USA; ^4^ NMS Labs Horsham Pennsylvania USA; ^5^ Faculty of Pharmaceutical Sciences University of São Paulo São Paulo SP Brazil; ^6^ Faculdade de Ciências Farmacêuticas Universidade Estadual de Campinas (UNICAMP) Campinas SP Brazil

**Keywords:** LC–MS/MS, micro‐QuEChERS, nitazenes, plasma, toxicology

## Abstract

Nitazenes are highly potent synthetic opioids of increasing concern in clinical and forensic toxicology, thus underscoring the need for sensitive and selective analytical methods for biological specimens. This study describes the development and validation of an LC–MS/MS method for the quantification of nitazene analogs and related metabolites in plasma using micro‐QuEChERS sample preparation. The validated concentration range was 0.05–20 ng/mL, with coefficients of determination *R* ^2^ > 0.99. The limit of detection (LOD) was 0.025 ng/mL, and the lower limit of quantification (LLOQ) was 0.05 ng/mL. Bias ranged from −2.6% to 15.7%, within‐run precision from 3.4% to 12.7%, and between‐run precision from 4.4% to 14.9%, as evaluated at three quality control levels. Matrix effects and recovery were analyte‐dependent but compatible with satisfactory overall validation performance. No interferences or carryover were observed. Method applicability was demonstrated in an authentic intoxication case, in which *N*‐pyrrolidino protonitazene was quantified at 1.4 ng/mL, representing, to our knowledge, the first clinical case reported in Brazil. The case was clinically characterized by severe opioid toxicity requiring repeated naloxone administration. The proposed method combined low sample volume, simple sample preparation, and suitable analytical performance for targeted analysis of emerging nitazene compounds. These findings support the use of micro‐QuEChERS and LC–MS/MS as a practical approach for targeted nitazene quantification in clinical and forensic toxicology.

## Graphical abstract

Micro‐QuEChERS sample preparation coupled to LC‐QQQ‐MS enabled sensitive quantification of nitazene analogs in 100 μL of plasma. The validated method provided low detection limits and was successfully applied to an authentic intoxication case involving *N*‐pyrrolidino protonitazene, supporting clinical and forensic toxicology workflows.

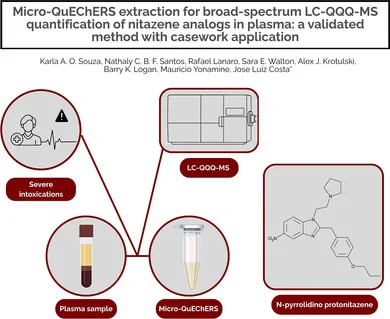

## 1 Introduction

Nitazenes are a rapidly expanding class of highly potent synthetic opioids that have emerged in illicit drug markets and become a major concern in clinical and forensic toxicology. Although originally synthesized decades ago as potential analgesics, these 2‐benzylbenzimidazole derivatives have recently been linked to an increasing number of severe intoxications and deaths across North America, Europe, Australia, and, more recently, South America. Their circulation is particularly concerning because they are frequently encountered in heroin‐like products, counterfeit tablets, or mixed drug preparations rather than being knowingly consumed as nitazenes, which increases the risk of unintentional exposure [1, 2, 3].

From a toxicological perspective, nitazenes may cause severe opioid toxicity even at very low concentrations. Their effects are usually consistent with the classic opioid toxidrome, especially sedation, miosis, and respiratory depression. Recent clinical reports indicate that naloxone remains effective for nitazene poisoning, although repeated doses or prolonged observation may be required in some cases. In addition to unintentional exposure, many reported intoxications involve multiple substances, which complicates toxicological interpretation and limits the definition of concentration–effect relationships or clinically meaningful thresholds for individual analogs. Together, these factors highlight the need for reliable laboratory methods to confirm and quantify nitazenes in biological samples [1, 2, 4].

The analytical complexity is further compounded by the structural diversity of this class. Small structural modifications in the nitazene core can markedly affect μ‐opioid receptor activity, and closely related analogs may differ in potency, toxicological relevance, and analytical behavior. Routine toxicological screening does not reliably detect nitazenes, and method development is complicated by closely related analogs and isomeric compounds, as well as by the limited availability of certified reference materials and spectral libraries. This is particularly relevant in analytical toxicology because the group now includes not only parent nitazenes but also *N*‐desethyl metabolites, desnitazenes, and pyrrolidino‐ or piperidinyl‐substituted compounds, all of which may be analytically relevant. Because many nitazenes are present at low concentrations in biological specimens, methods for this class must combine high sensitivity, selectivity, and sufficient flexibility to address a rapidly evolving group of chemically related substances [1, 5, 6].

Recent reviews have highlighted the need for robust quantitative methods for nitazenes in biological matrices [3, 5]. LC–MS/MS methods have been described in whole blood, dried blood spots, hair, and other biological matrices. These studies have demonstrated that sensitive quantitative analysis is feasible, but the available literature remains fragmented across matrices and analytical purposes.

Whole blood methods may offer broad applicability in forensic toxicology but are more affected by matrix‐related interference, whereas dried microsampling approaches and other miniaturized formats are often designed to simplify collection and handling. Plasma is also a relevant matrix for targeted analysis, particularly in clinical toxicology, and may provide a cleaner analytical background, but it still requires proper validation and careful definition of the analytes included in the method, especially when both parent compounds and related metabolites are considered [5, 7, 8, 9].

In this context, there is still a need for simple and validated quantitative methods that combine broad nitazene coverage, low sample volume requirements, and practical applicability to real toxicological casework. To our knowledge, the use of micro‐QuEChERS sample preparation for targeted quantitative analysis of nitazenes in plasma has not yet been described. Therefore, the aim of the present study was to develop and validate a targeted LC–MS/MS method for the quantification of nitazene analogs and selected related metabolites in plasma using micro‐QuEChERS sample preparation, and to demonstrate its applicability in an authentic intoxication case. The chemical structures of the analytes included in the present study are shown in Figure 1.

**FIGURE 1:**
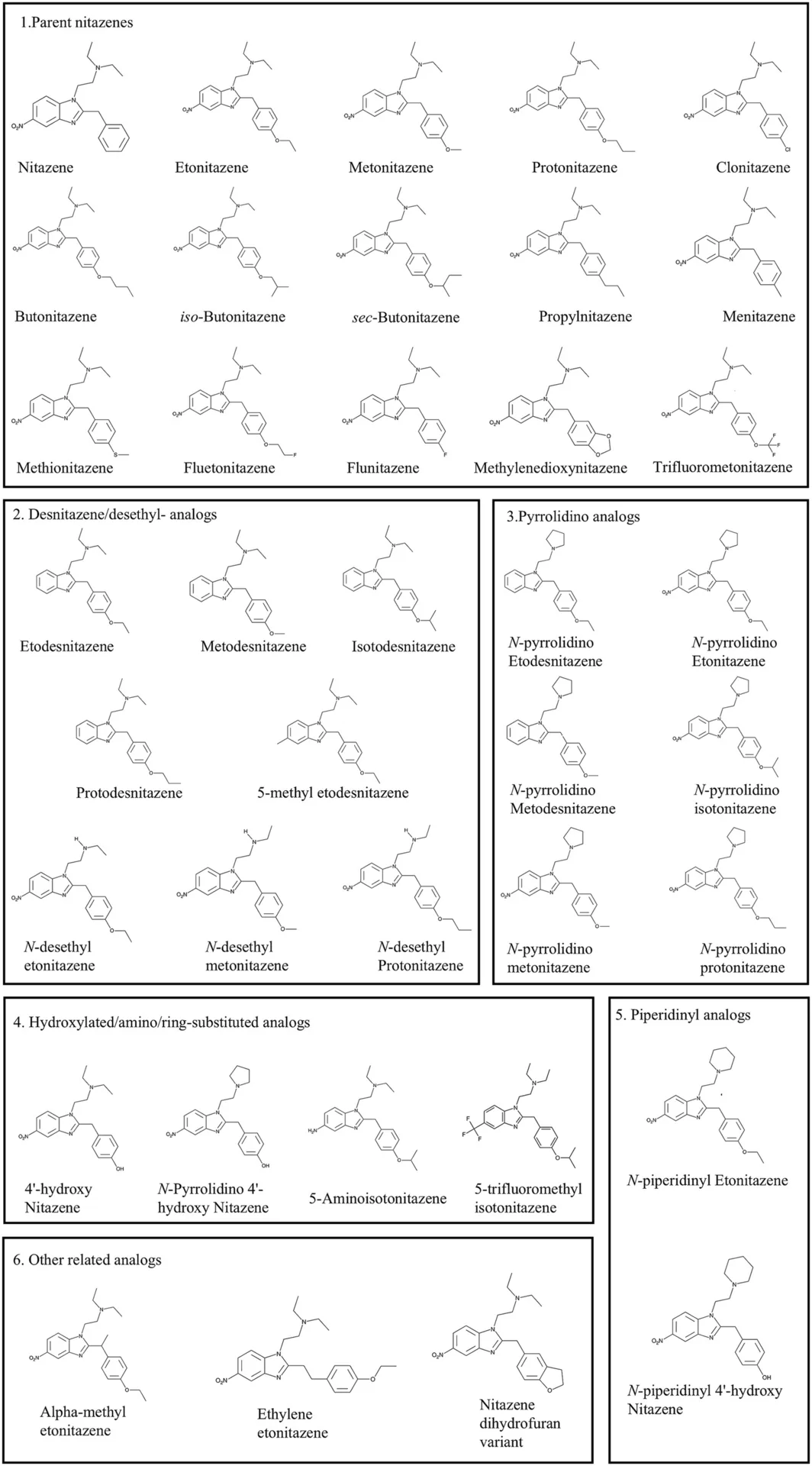
Chemical structures of the nitazene analogs and selected related metabolites included in the present study, organized according to structural subgroup.

## 2 Materials and Methods

### 2.1 Chemicals, Reagents, and Biological Matrices

Reference standards of nitazene analogs and related compounds including 4′‐hydroxy nitazene, 5‐aminoisotonitazene, 5‐methyl etodesnitazene, 5‐trifluoromethyl isotonitazene, alpha‐methyl etonitazene, butonitazene, clonitazene, ethylene etonitazene, etodesnitazene, etonitazene, fluetonitazene, flunitazene, *iso*‐butonitazene, isotodesnitazene, menitazene, methionitazene, methylenedioxynitazene, metodesnitazene, metonitazene, *N*‐desethyl etonitazene, *N*‐desethyl metonitazene, *N*‐desethyl protonitazene, nitazene, nitazene dihydrofuran variant, *N*‐piperidinyl 4′‐hydroxy nitazene, *N*‐piperidinyl etonitazene, *N*‐pyrrolidino 4′‐hydroxy nitazene, *N*‐pyrrolidino etodesnitazene, *N*‐pyrrolidino etonitazene, *N*‐pyrrolidino isotonitazene, *N*‐pyrrolidino metodesnitazene, *N*‐pyrrolidino metonitazene, *N*‐pyrrolidino protonitazene, propylnitazene, protodesnitazene, protonitazene, pyrrolidino variant etonitazene, sec‐butonitazene, and trifluorometonitazene were obtained from Cayman Chemical (Ann Arbor, Michigan, United States). Fentanyl‐d5 was used as the internal standard (IS) and was purchased from Cayman Chemical (Ann Arbor, Michigan, United States LC‐MS grade methanol, acetonitrile, formic acid, and ammonium formate were purchased from Merck (Darmstadt, Germany). Anhydrous magnesium sulfate (MgSO_4_) and sodium acetate (NaAc), used for the micro‐QuEChERS extraction, were purchased from Sigma‐Aldrich (St. Louis, Missouri, United States). Ultrapure water was generated using a Milli‐Q purification system (Millipore, Milford, Massachusetts, United States).

Blank human plasma used for method validation was obtained from Campinas Blood Center (Campinas, SP, Brazil). Plasma samples were anonymized and confirmed to be free of the target analytes before use.

### 2.2 LC–MS/MS Conditions

Analyses were performed using a UHPLC system coupled to an LCMS‐8060 triple quadrupole mass spectrometer (Shimadzu, Kyoto, Japan). Chromatographic separation was achieved on a Kinetex C18 column (3.0 mm × 50 mm, 2.6 μm) (Phenomenex, Torrance, California, United States) maintained at 40 °C. The mobile phase consisted of ultrapure water (A) and methanol (B), both containing 0.1% formic acid and 2 mmol/L ammonium formate. The gradient program was as follows: 0–1.0 min, 5% B; linear increase to 100% B at 9.0 min; 9.0–11.3 min, 100% B; return to 5% B at 11.4 min; and re‐equilibration at 5% B until 13.5 min. The flow rate was 0.30 mL/min, the injection volume was 5 μL, and the autosampler temperature was maintained at 10 °C.

Data were acquired in multiple reaction monitoring (MRM) mode, using two transitions per analyte. The mass spectrometer was operated using an electrospray ionization (ESI) source in positive ion mode. The source conditions were as follows: heating block temperature, 400 °C; capillary voltage, 4.0 kV; nebulizing gas flow (N_2_), 3 L/min; desolvation line temperature, 250 °C; drying gas flow (N_2_), 10 L/min; heating gas flow (air), 10 L/min; interface temperature, 300 °C; and argon collision‐induced dissociation (CID) gas pressure, 270 kPa. Data acquisition and processing were performed using LabSolutions 5.10 and Insight 3.8 software (Shimadzu, Kyoto, Japan). Collision energies, m/z transitions, and retention times are summarized in Table 1.

**TABLE 1:** MRM transitions and retention times for all targeted analytes, with quantifier transitions underlined.

Analyte	MRM transitions	MRM ratio (%)	Ion ratio interval (%)	Q1 pre bias (V)	CE (eV)	Q3 pre bias (V)	Retention time (min)
4′‐Hydroxy nitazene	369.2 ≥ 100.0 369.2 > 72.0	17.3	12.1–22.5	−14.0 −14.0	−22.0 −40.0	−18.0 −28.0	4.6
5‐Aminoisotonitazene	381.2 ≥ 100.0 381.2 > 72.0	13.3	9.3–17.3	−11.0 −15.0	−23.0 −44.0	−17.0 −26.0	4.2
5‐Methyl etodesnitazene	366.2 ≥ 100.0 366.2 > 44.0	15.2	11.4–19.0	−19.0 −14.0	−21.0 −55.0	−17.0 −16.0	5.4
5‐Trifluoromethyl isotonitazene	434.2 ≥ 100.0 434.2 > 72.0	14.7	11.0–18.4	−13.0 −22.0	−23.0 −48.0	−17.0 −29.0	7.1
Alpha‐methyl etonitazene	411.2 ≥ 100.0 411.2 > 72.0	18.3	12.8–23.7	−10.0 −21.0	−24.0 −43.0	−17.0 −27.0	6.3
Butonitazene	425.3 ≥ 100.0 425.3 > 72.0	16.5	11.6–21.4	−10.0 −16.0	−25.0 −46.0	−17.0 −29.0	7.1
Clonitazene	387.2 ≥ 100.0 387.2 > 72.0	18.3	13.7–22.9	−11.0 −11.0	−22.0 −40.0	−18.0 −12.0	6.1
Ethylene etonitazene	411.2 ≥ 100.0 411.2 > 72.0	15.9	11.9–19.9	−16.0 −21.0	−24.0 −49.0	−17.0 −27.0	6.4
Etodesnitazene	352.2 ≥ 100.0 352.2 > 44.0	16.3	12.2–20.4	−18.0 −10.0	−21.0 −53.0	−17.0 −18.0	5.1
Etonitazene	397.2 ≥ 100.1 397.2 > 72.0	14.2	9.9–18.4	−12.0 −20.0	−22.0 −42.0	−17.0 −29.0	6.0
Fluetonitazene	415.2 ≥ 100.0 415.2 > 72.0	17.8	12.4–23.1	−16.0 −12.0	−25.0 −44.0	−17.0 −29.0	5.5
Flunitazene	371.2 ≥ 100.0 371.2 > 44.0	23.4	17.5–29.2	−11.0 −11.0	−20.0 −42.0	−17.0 −29.0	5.6
*Iso*‐butonitazene	425.3 ≥ 100.0 425.3 > 72.0	16.3	12.2–20.4	−13.0 −21.0	−24.0 −46.0	−17.0 −29.0	7.1
Isotodesnitazene	366.2 ≥ 100.1 366.2 > 44.1	11.5	8.1–15.0	−11.0 −19.0	−23.0 −51.0	−18.0 −16.0	5.6
Menitazene	367.2 ≥ 100.2 367.2 > 44.1	16.4	11.5–21.4	−14.0 −17.0	−23.0 −51.0	−10.0 −16.0	5.9
Methionitazene	399.2 ≥ 100.0 399.2 > 72.0	20.0	15.0–25.1	−12.0 −12.0	−22.0 −45.0	−16.0 −12.0	6.1
Methylenedioxynitazene	397.2 ≥ 100.0 397.2 > 72.0	14.7	10.3–19.1	−15.0 −12.0	−21.0 −41.0	−17.0 −28.0	5.4
Metodesnitazene	338.2 ≥ 100.0 338.2 > 44.0	14.7	10.2–19.0	−18.0 −10.0	−21.0 −54.0	−18.0 −16.0	4.4
Metonitazene	383.2 ≥ 100.0 383.2 > 72.0	21.6	15.1–28.1	−11.0 −19.0	−22.0 −41.0	−17.0 −28.0	5.5
*N*‐desethyl etonitazene	369.2 ≥ 72.0 369.2 > 298.0	18.2	13.6–22.7	−11.0 −18.0	−21.0 −20.0	−27.0 −19.0	6.0
*N*‐desethyl metonitazene	355.2 ≥ 72.0 355.2 > 284.0	16.0	12.0–20.0	−14.0 −18.0	−21.0 −20.0	−29.0 −30.0	5.5
*N*‐desethyl protonitazene	383.2 ≥ 72.0 383.2 > 44.0	19.1	14.3–23.9	−11.0 −11.0	−50.0 −22.0	−24.0 −30.0	6.6
Nitazene	353.2 ≥ 100.0 353.2 > 44.0	16.4	11.4–21.2	−18.0 −13.0	−24.0 −55.0	−17.0 −17.0	5.4
Nitazene dihydrofuran variant	395.2 ≥ 100.0 395.2 > 72.0	16.8	11.7–21.8	−20.0 −15.0	−21.0 −46.0	−18.0 −28.0	5.5
*N*‐piperidinyl 4′‐hydroxy nitazene	381.2 ≥ 112.0 381.2 > 107.0	7.2	5.4–9.1	−18.0 −15.0	−26.0 −47.0	−19.0 −20.0	4.7
*N*‐piperidinyl etonitazene	409.2 ≥ 112.0 409.2 > 107.0	8.5	6.4–10.6	−10.0 −12.0	−25.0 −50.0	−18.0 −19.0	6.1
*N*‐pyrrolidino 4′‐hydroxy nitazene	367.0 ≥ 98.0 367.0 > 107.0	7.7	3.9–11.6	−14.0 −14.0	−24.0 −37.0	−17.0 −16.0	4.5
*N*‐pyrrolidino etodesnitazene	350.2 ≥ 98.1 350.2 > 56.0	13.1	9.2–17.1	−18.0 −13.0	−22.0 −53.0	−17.0 −22.0	4.9
*N*‐pyrrolidino etonitazene	395.2 ≥ 98.0 395.2 > 56.0	15.1	10.6–19.6	−12.0 −12.0	−22.0 −52.0	−17.0 −21.0	6.0
*N*‐pyrrolidino isotonitazene	409.2 ≥ 98.0 409.2 > 56.0	16.4	12.3–20.5	−20.0 −10.0	−24.0 −54.0	−17.0 −22.0	6.3
*N*‐pyrrolidino metodesnitazene	336.2 ≥ 98.0 336.2 > 56.0	13.9	9.7–18.0	−17.0 −17.0	−21.0 −54.0	−17.0 −21.0	4.2
*N*‐pyrrolidino metonitazene	381.2 ≥ 98.1 381.2 > 56.1	18	12.6–23.4	−14.0 −19.0	−24.0 −54.0	−18.0 −21.0	5.4
*N*‐pyrrolidino protonitazene	409.2 ≥ 98.0 409.2 > 56.0	12.2	8.5–15.9	−21.0 −15.0	−25.0 −55.0	−17.0 −23.0	6.5
Propylnitazene	395.3 ≥ 100.0 395.3 > 72.0	19.5	14.6–24.3	−15.0 −12.0	−22.0 −42.0	−18.0 −27.0	6.9
Protodesnitazene	366.2 ≥ 100.0 366.2 > 44.0	11.4	8.0–14.8	−11.0 −11.0	−22.0 −54.0	−17.0 −16.0	5.9
Protonitazene	411.2 ≥ 100.0 411.2 > 72.0	17.5	12.2–22.7	−10.0 −12.0	−23.0 −41.0	−17.0 −28.0	6.6
Pyrrolidino variant etonitazene	409.2 ≥ 112.0 409.2 > 84.0	22.1	16.6–27.6	−12.0 −12.0	−24.0 −47.0	−19.0 −14.0	6.2
*Sec*‐butonitazene	425.3 ≥ 100.1425.3 > 72.1	15.4	10.8–20.0	−10.0 −12.0	−21.0 −45.0	−18.0 −27.0	6.9
Trifluorometonitazene	437.2 ≥ 100.0 437.2 > 72.0	20.0	14.0–25.9	−16.0 −13.0	−23.0 −43.0	−17.0 −12.0	6.7
Fentanyl‐D_5_	342.3 ≥ 105.1 342.3 > 137.1	28.3	21.2–35.4	−12.0 −12.0	−39.0 −34.0	−12.0 −28.0	5.7

Abbreviation: CE, collision energy.

### 2.3 Preparation of Stock Solutions, Calibration Standards, and Quality Control Samples

Stock solutions of all analytes were prepared in methanol at 10 μg/mL and further diluted in the same solvent to obtain the corresponding working solutions. Calibration working solutions were prepared to yield final plasma concentrations of 0.05, 0.1, 1, 2, 5, 10, 15, and 20 ng/mL after fortification of the blank plasma with 10 μL of the corresponding working solution. Quality control (QC) working solutions were prepared to obtain final plasma concentrations of 0.15, 8, and 16 ng/mL, corresponding to the low (LQC), medium (MQC), and high QC (HQC) levels, respectively, also by addition of 10 μL of working solution. Fentanyl‐d_5_ was prepared in methanol at a concentration of 50 ng/mL and used as a surrogate internal standard for the full analyte panel, as isotopically labeled nitazene analogs were not available for all target compounds during method development. Calibration and QC working solutions were prepared at 10‐fold the target final concentration to account for dilution during sample fortification. All solutions were stored at −20 °C until analysis.

### 2.4 Extraction Procedure

An aliquot of 100 μL of blank or fortified human plasma was transferred to a 2 mL polypropylene tube, and 10 μL of the IS solution (fentanyl‐d_5_, 50 ng/mL) was added. Subsequently, 400 μL of acetonitrile and 100 mg of QuEChERS extraction salts (NaAc/MgSO₄, 1:4, w/w) were added. The samples were agitated at 2500 rpm for 5 min and then centrifuged at 14,000 rpm for 5 min. The supernatant was collected and evaporated to dryness under a nitrogen stream at 40 °C. The dried residue was reconstituted in 100 μL of methanol, and 5 μL was injected into the LC–MS/MS system.

### 2.5 Method Validation

The method was validated according to ANSI/ASB Standard 036 (2019) [10] guidelines for forensic toxicology. The validation included assessment of selectivity, carryover, linearity, limit of detection (LOD), lower limit of quantification (LLOQ), precision, bias, matrix effect, recovery, and stability. Calibration curves were evaluated over five independent analytical runs using 1/*x* ^2^ weighted linear regression. Precision and bias were assessed in triplicate at three QC levels (0.15, 8, and 16 ng/mL), corresponding to the LQC, MQC, and HQC levels, respectively. All acceptance criteria were established according to the guideline.

### 2.6 Selectivity

Selectivity was evaluated by analyzing 10 independent sources of blank human plasma. These samples were processed according to the proposed extraction protocol without the addition of the IS to assess endogenous interferents at the monitored MRM transitions and retention times of the target analytes. Additional blank plasma samples were also analyzed with the addition of the IS only to confirm the absence of interfering peaks. Selectivity was further assessed in the presence of common exogenous compounds not included in this method, including other opioid analogs, new psychoactive substances, and other commonly encountered drugs, to evaluate the potential for analytical interference. A complete list of compounds included is provided in the Supplementary Material (Tables S2 and S3).

### 2.7 Carryover

Carryover was assessed by injecting extracted blank plasma samples immediately after the highest calibration standard (20 ng/mL). Residual signal was evaluated at the retention times and monitored MRM transitions of the target analytes and the internal standard. Carryover was considered present when the blank sample met the identification criteria for any analyte detected in the preceding high‐concentration injection.

### 2.8 Linearity

Linearity was assessed using pooled blank plasma samples fortified at eight concentration levels (0.05, 0.1, 1, 2, 5, 10, 15, and 20 ng/mL). Calibration curves were analyzed over five independent analytical runs to evaluate the linear response of each analyte. Weighted linear regression (1/*x* ^2^) was applied using the analyte‐to‐internal standard peak area ratio. Calibrators were considered acceptable when quantified within ±20% of the nominal concentration.

### 2.9 Limits of Detection and Quantification

LOD and LLOQ were determined using fortified plasma samples prepared at decreasing concentrations. The LOD was defined as the lowest concentration at which the analyte could be reliably identified, meeting all identification criteria and producing a signal‐to‐noise (S/N) ratio of at least 3:1. The LLOQ was defined as the lowest calibrator meeting the method identification criteria, producing a S/N ratio greater than 10:1, and showing acceptable quantitative performance (bias and precision within ±20%).

### 2.10 Precision and Bias

Precision and bias were assessed by analyzing fortified pooled plasma samples in triplicate at three concentration levels (0.15, 8, and 16 ng/mL), corresponding to the LQC, MQC, and HQC samples, over five consecutive days (*n* = 15 per level). Bias was calculated as the percentage difference between the mean measured concentration at each level and the corresponding nominal concentration. Within‐run and between‐run precision were calculated as the relative standard deviation (%RSD) using one‐way analysis of variance (ANOVA). Acceptance criteria were defined as %RSD ≤ 20% for precision and bias within ±20% of the nominal concentration.

### 2.11 Matrix Effects and Recovery

Matrix effect and recovery were evaluated at LQC (0.15 ng/mL) and HQC (16 ng/mL) using independent sources of blank plasma. To assess the matrix effect, samples were extracted and fortified after extraction with the analyte solutions at the corresponding concentration levels. The absolute peak areas obtained for these post‐extraction samples were compared with those obtained for neat solutions prepared at equivalent concentrations. Matrix effect was expressed as the percentage of signal suppression or enhancement caused by the plasma matrix.

For recovery, blank plasma samples were fortified with the analyte solutions before extraction and processed according to the proposed procedure. The absolute peak areas obtained for these samples were compared with those obtained for post‐extraction fortified samples at the same concentration levels. Recovery was expressed as the percentage of analyte recovered after extraction.

### 2.12 Stability

Stability was evaluated in triplicate at the LQC and HQC levels under two conditions: autosampler stability and freeze–thaw stability. For autosampler stability, extracted samples were stored in the autosampler at 10 °C for 24 h prior to reanalysis and compared with freshly prepared controls. Freeze–thaw stability was evaluated using fortified plasma samples stored at −20 °C and subjected to 1, 3, and 5 freeze–thaw cycles before extraction and analysis, allowing complete thawing at room temperature prior to each cycle. Stability after freeze–thaw was assessed by comparing analyte‐to‐internal standard peak area ratios with those obtained for freshly prepared controls.

### 2.13 Application to an Authentic Intoxication Case

To demonstrate the applicability of the method, the validated workflow was applied to a sample obtained from an authentic intoxication case assisted by the Campinas Poison Control Center.

Additional toxicological screening was performed as part of the routine clinical toxicology workflow. Plasma was screened for other psychoactive substances by LC–MS/MS, and ethanol was determined by headspace gas chromatography. The corresponding urine sample was also qualitatively analyzed for nitazene‐related metabolites.

## 3 Results and Discussion

### 3.1 Chromatographic Performance and Selectivity

The LC–MS/MS conditions were suitable for the target analysis of nitazene analogs in plasma. This is particularly relevant because the rapid expansion of this class in the illicit drug market has introduced numerous structurally related analogs and positional isomers, which frequently exhibit similar chromatographic and mass spectrometric behavior, making their analytical discrimination difficult [5]. In this context, the proposed method provided a practical balance between analytical scope, sensitivity, and routine applicability for toxicological analysis, while maintaining a simple sample‐preparation procedure based on micro‐QuEChERS. The monitored MRM transitions, ion ratio intervals, and retention times are summarized in Table 1.

A relevant limitation was observed for the isomeric pair butonitazene and *iso*‐butonitazene. Under the established chromatographic conditions, both compounds coeluted and shared the same monitored transitions and ion ratio criteria (Table 1), which prevented their unambiguous individual discrimination. *Sec*‐butonitazene, which belongs to the same structural isomeric group, eluted earlier (RT 6.9 min) than butonitazene and *iso*‐butonitazene (RT 7.1 min). Inspection of reference‐standard chromatograms showed chromatographic separation between the main analytical peak of *sec*‐butonitazene and those of butonitazene and iso‐butonitazene under the present LC conditions. This finding is consistent with recent literature showing that the butonitazene group remains analytically challenging even in more advanced workflows. Wachełko et al. [11] showed that the butonitazene group (butonitazene, *iso*‐butonitazene, and *sec*‐butonitazene) presents greater challenges during method development than other isomers. In their UHPLC–MS/MS method, the separation of these compounds required dedicated stationary phases, particularly pentafluorophenyl (PFP) and extended gradients (22 min), whereas C18 columns (with 40 min gradient) did not achieve complete resolution of butonitazene and *iso*‐butonitazene without signal overlap. Furthermore, Hollerbach et al. [12] reported that even advanced analytical platforms, such as ion mobility‐Orbitrap mass spectrometry (SLIM‐IMS‐MS), achieved only partial differentiation of this specific group, indicating that these analogs remain difficult to resolve even with high‐resolution approaches. Accordingly, the present method should not be interpreted as an isomer‐resolving workflow for this specific pair, but rather as a practical targeted assay for broad nitazene quantification in plasma.

From a practical perspective, the proposed workflow was suitable for routine toxicological analysis. The extraction protocol required only 100 μL of plasma and a simple micro‐QuEChERS procedure. Given the high potency of many nitazenes and the low concentrations at which they may be detected in biological specimens, efficient and reliable sample preparation remains essential, alongside adequate instrumental sensitivity [1, 2, 13]. Recent studies on nitazene analysis have also highlighted the value of simplified sample preparation and reduced sample volume to improve routine applicability in clinical and forensic laboratories [7, 8]. For example, Schüller et al. [7] used liquid‐phase microextraction (LPME) with 120 μL of whole blood, whereas Ververi et al. [8] described a dried blood spot (DBS) method using only 10 μL of sample. In this context, the present micro‐QuEChERS approach offers a simple and low‐volume alternative for nitazene analysis. The chromatographic and extraction conditions also allowed the inclusion of a broad analytical panel during method development, and this scope may be expanded as new nitazene analogs and reference standards become available.

### 3.2 Method Validation

#### 3.2.1 Selectivity, Carryover, and Linearity

Selectivity was demonstrated for all analytes under the proposed conditions. No endogenous or exogenous interferences were observed, supporting the specificity of the method. Carryover was not observed for any of the monitored analytes or for the internal standard when blank samples were injected after the highest calibration level. The method also showed satisfactory linearity over the validated concentration range of 0.05–20 ng/mL. Calibration curves analyzed over five independent runs produced coefficients of determination (*R* ^2^) greater than 0.99 for all analytes, and all calibrators met the predefined acceptance criteria.

The calibration range is consistent with recent LC–MS/MS methods reported for nitazenes in biological matrices [8, 14]. The validated range of 0.05–20 ng/mL also covers many concentrations reported in authentic nitazene intoxications and deaths, although comparisons across matrices and case types should be interpreted cautiously. For example, concentrations around 1–2 ng/mL have been reported for isotonitazene and *N*‐desethyl isotonitazene, while values around 5–6 ng/mL have been described for metonitazene and etonitazepyne. At the same time, protonitazene‐related cases may show a wider concentration range, and some severe postmortem cases may exceed the upper limit of the validated interval [2, 14, 15, 16, 17, 18, 19, 20]. In this context, the present method provides a validated linear range compatible with a substantial proportion of reported nitazene‐positive cases.

#### 3.2.2 Sensitivity, Precision, and Bias

The method showed suitable sensitivity for the targeted analytes. Because nitazenes are highly potent and may be present at very low concentrations in biological specimens, analytical methods for this class require low detection and quantification limits while maintaining reliable quantitative performance [1]. Despite the use of the miniaturized micro‐QuEChERS workflow and only 100 μL of plasma, the method achieved an LOD of 0.025 ng/mL and an LLOQ of 0.05 ng/mL, which are compatible with the concentration range reported for a substantial proportion of authentic nitazene‐positive cases. This is relevant because reduced sample volume and simplified extraction workflows may compromise sensitivity in biological matrices.

Direct comparison with previously published methods should be interpreted cautiously because of differences in matrix, sample preparation, analyte scope, and study purpose. Nevertheless, the sensitivity achieved in the present study compares favorably with previously published nitazene methods. Walton et al. [14] reported an LC–MS/MS method in human whole blood using a 500 μL sample volume and liquid–liquid extraction (LLE), achieving an LOD of 0.1 ng/mL and an LOQ of 0.5 ng/mL. Ververi et al. [8] described a DBS method requiring only 10 μL of blood, but with a higher calibration range in the 1–50 ng/mL range and LOD values between 0.3 and 0.5 ng/mL. Recently, Wachełko et al. [11] reported LOQs of 0.01 ng/mL for most nitazene analogs and 0.05 ng/mL for flunitazene; however, their method also required 500 μL of whole blood extracted with LLE. In this regard, the present workflow provides a useful balance between sensitivity, analytical scope, and reduced sample‐volume requirements. Representative LC–MS/MS chromatograms at the LLOQ are shown in Figure S1.

Precision and bias were also satisfactory for the validated analytes. Bias ranged from −2.64% to 15.67%, within‐run precision ranged from 3.36% to 12.74%, and between‐run precision ranged from 4.35% to 14.88% (Table 2). These values were within the predefined acceptance criteria and indicate that the proposed method provided reliable quantitative performance over the evaluated concentration range.

**TABLE 2:** Method validation results for within‐run and between‐run imprecisions, biases, matrix effects, and recoveries for analyses of nitazenes in plasma samples using LC–MS/MS.

	Bias (%)			Within‐run imprecision (%RSD)			Between‐run imprecision (%RSD)			Matrix effect (%)		Recovery (%)	
Analyte	LQC	MQC	HQC	LQC	MQC	HQC	LQC	MQC	HQC	LQC	HQC	LQC	HQC
4′‐Hydroxy nitazene	4.67	−2.06	−1.50	6.58	9.14	3.97	6.02	9.62	8.51	33	−7	115	144
5‐Aminoisotonitazene	6.67	−2.48	−0.67	10.70	10.50	5.47	9.10	11.48	11.74	82	20	94	132
5‐Methyl etodesnitazene	8.67	2.88	9.61	11.14	5.11	3.93	11.63	5.34	8.70	101	64	86	78
5‐Trifluoromethyl isotonitazene	10.00	4.63	8.68	10.50	5.22	4.13	11.61	5.91	8.16	48	32	85	75
Alpha‐methyl etonitazene	2.33	−2.31	−0.29	4.68	9.39	4.39	6.76	9.18	6.74	17	−13	85	107
Butonitazene	8.33	1.62	3.29	5.21	8.85	3.88	5.82	8.89	8.12	25	−11	81	110
Clonitazene	13.67	3.28	5.17	11.71	4.67	3.68	11.11	4.60	7.58	56	31	89	77
Ethylene etonitazene	12.00	3.50	7.50	10.25	5.22	3.58	10.37	6.49	7.27	57	40	88	76
Etodesnitazene	11.33	3.57	8.45	8.68	5.69	3.71	9.39	6.38	8.84	99	84	86	78
Etonitazene	8.33	2.77	1.04	6.20	6.36	6.05	6.80	7.29	9.15	52	30	89	80
Fluetonitazene	6.67	3.26	1.79	6.25	5.46	5.75	5.98	8.54	8.25	54	33	89	81
Flunitazene	8.33	−0.60	−0.43	10.02	9.87	3.60	8.92	8.99	5.52	16	−13	84	105
*Iso*‐butonitazene	9.33	2.04	5.98	11.13	4.86	3.67	10.18	5.70	8.58	146	100	85	82
Isotodesnitazene	10.00	6.07	4.54	8.50	6.20	5.41	8.79	6.57	7.78	12	−14	87	101
Menitazene	6.67	1.96	0.15	6.85	7.14	6.17	7.13	8.05	10.52	43	25	91	83
Methionitazene	15.67	3.48	5.30	12.29	4.53	3.98	13.79	5.04	8.93	50	27	88	78
Methylenedioxynitazene	6.33	−0.97	−0.21	6.00	9.63	3.53	5.87	9.31	7.34	36	−25	88	99
Metodesnitazene	8.67	4.49	9.79	10.68	5.43	3.84	10.77	5.21	7.89	22	−12	83	106
Metonitazene	4.33	−1.79	0.87	6.01	10.62	3.77	8.32	10.34	7.36	153	83	93	105
*N*‐desethyl etonitazene	15.67	5.03	5.38	9.79	5.58	4.54	9.17	6.23	8.70	74	−3	92	106
*N*‐desethyl metonitazene	14.33	4.31	6.26	12.16	5.40	4.85	14.88	6.11	7.55	38	−23	110	175
*N*‐desethyl protonitazene	6.67	−0.57	−0.78	8.76	9.65	4.10	8.88	9.99	8.01	69	11	84	109
Nitazene	4.33	−2.01	−1.13	5.02	9.39	3.36	6.45	8.81	6.21	15	−13	87	108
Nitazene dihydrofuran variant	7.67	2.66	0.69	6.92	5.33	6.27	7.11	8.73	9.27	57	29	87	79
*N*‐piperidinyl 4′‐hydroxy nitazene	14.67	4.91	8.56	11.43	5.20	4.28	11.64	6.51	8.79	77	17	91	125
*N*‐piperidinyl etonitazene	13.67	5.40	8.67	12.74	4.85	4.09	14.56	5.38	7.77	7	−14	84	100
*N*‐pyrrolidino 4′‐hydroxy nitazene	10.00	−0.74	−0.62	6.16	8.12	4.29	5.47	7.14	6.13	24	−11	79	106
*N*‐pyrrolidino etodesnitazene	6.67	−0.09	1.20	4.84	8.76	3.60	4.35	8.62	5.31	13	−14	86	107
*N*‐pyrrolidino etonitazene	4.67	−1.61	0.01	5.93	8.51	3.76	5.85	8.38	6.56	−6	−6	101	102
*N*‐pyrrolidino isotonitazene	12.00	5.68	9.29	11.29	4.80	4.35	13.96	4.79	6.91	5	2	93	96
*N*‐pyrrolidino metodesnitazene	3.00	−1.64	−0.29	5.22	6.56	3.38	4.73	6.92	5.64	−13	−15	107	113
*N*‐pyrrolidino metonitazene	8.33	3.39	2.00	8.14	5.11	5.34	8.24	9.16	9.07	17	5	84	93
*N*‐pyrrolidino protonitazene	4.00	0.76	−1.99	8.76	8.55	7.14	9.75	10.64	12.11	7	−5	103	104
Propylnitazene	11.00	1.93	6.21	9.46	5.24	3.55	9.68	6.71	9.30	−10	−14	105	108
Protodesnitazene	5.67	0.14	3.10	5.34	8.79	4.01	5.62	9.05	6.70	−5	−13	107	113
Protonitazene	6.00	1.23	−2.64	8.36	8.39	7.09	9.85	11.70	14.09	−12	−10	115	125
Pyrrolidino variant etonitazene	3.33	−2.13	−1.15	5.77	9.47	4.06	5.07	8.51	5.93	−21	−19	95	97
*Sec*‐butonitazene	8.00	6.65	3.25	8.80	8.98	7.43	8.54	9.33	10.64	3	8	92	93
Trifluorometonitazene	9.33	7.87	3.28	9.51	8.53	6.98	9.28	10.10	11.35	9	12	92	93

#### 3.2.3 Matrix Effect and Recovery

Matrix effect and recovery results are summarized in Table 2. Matrix effect ranged from −21% to 153% at the LQC level and from −25% to 100% at the HQC level, indicating that both ion suppression and ion enhancement were observed. Some analytes showed more pronounced matrix‐related effects. For example, metonitazene exhibited marked signal enhancement at LQC (153%), whereas methylenedioxynitazene showed the most pronounced suppression at HQC (−25%) among the analytes. This pattern is consistent with previous LC–MS/MS methods developed for nitazenes. Pardi et al. [21] reported ion enhancement up to 154% for metonitazene in blood, while Walton et al. [14] also described matrix enhancement for all evaluated nitazenes, including 174% for metonitazene, without compromising overall assay performance. Schüller et al. [7] likewise observed ion enhancement up to 233% for nitazenes during LPME. However, such pronounced matrix effects do not preclude satisfactory validation performance as long as appropriate matrix‐matched calibration and internal standardization are applied. This was confirmed in the present study, as reflected by the acceptable precision and bias values obtained for the validated analytes.

A single internal standard (fentanyl‐d5) was used in this study as a surrogate approach for a broad multi‐analyte panel. Although isotopically labeled nitazene analogs would provide more appropriate compensation for analyte‐specific extraction variability and matrix effects, labeled standards covering the full analyte panel were not available at the time of method development. In addition, the inclusion of one or more labeled nitazene analogs would improve correction for selected compounds but would still not provide analyte‐matched compensation across the structurally diverse 39‐analyte panel. Therefore, the present approach should be interpreted with caution, as a non‐matched internal standard may not fully correct specimen‐to‐specimen matrix variation in routine casework. To mitigate this limitation, matrix‐matched calibration was used, and method performance was individually assessed for each analyte through validation of precision, bias, matrix effect, and recovery. Accordingly, the method was considered suitable for the validated panel, while acknowledging that the use of analyte‐matched labeled internal standards would represent an important improvement for future applications.

Apparent extraction recovery ranged from 79% to 115% at the low QC level and from 75% to 175% at the high QC level. N‐desethyl metonitazene showed the highest apparent extraction recovery at the high QC level (175%), while 5‐trifluoromethyl isotonitazene showed the lowest value at the same level (75%). Because recovery was calculated by comparing pre‐extraction fortified samples with post‐extraction fortified matrix extracts, matrix‐related ion suppression or enhancement would be expected to largely cancel out in this comparison. Therefore, values above 100% were not interpreted as true absolute extraction yields, but rather as apparent recovery estimates. Although true absolute extraction recovery cannot exceed 100%, bioanalytical validation guidance emphasizes that recovery should be consistent and reproducible and should not compromise precision or bias. In the present study, despite the elevated apparent recovery observed for *N*‐desethyl metonitazene at the high QC level, the method met the predefined acceptance criteria for precision and bias, supporting its suitability for quantitative application within the validated conditions. Overall, the proposed micro‐QuEChERS method provided adequate apparent extraction performance for a structurally diverse nitazene panel in plasma.

#### 3.2.4 Stability

Stability results are summarized in Table 3. Autosampler stability after 24 h at 10 °C was generally acceptable. For most analytes, deviations remained within ±20% at both QC levels. Exceptions were limited to *N*‐desethyl metonitazene at LQC (+27.6%).

**TABLE 3:** Stability results for the nitazenes including autosampler stability after 24 h at 10 °C and freeze–thaw stability after 1, 3, and 5 cycles, evaluated at low‐ and high‐quality control levels.

	Freeze/thaw						Autosampler	
	LQC			HQC			24 h/10 °C	
Analyte	T1	T3	T5	T1	T3	T5	LQC	HQC
4′‐Hydroxy nitazene	25.9	11.2	3.7	11.0	6.6	2.7	−8.2	−12.5
5‐Aminoisotonitazene	−6.3	3.9	−29.1	−10.7	−33.9	−30.8	−6.1	−14.9
5‐Methyl etodesnitazene	1.6	−9.0	−7.9	8.3	−13.6	4.6	−13.3	−17.9
5‐Trifluoromethyl isotonitazene	6.7	−5.5	−5.5	15.1	−9.1	7.6	3.3	−5.8
Alpha‐methyl etonitazene	32.4	−0.5	−8.5	10.9	−1.7	−5.3	6.7	−8.3
Butonitazene	24.3	−2.2	−12.0	3.9	−11.5	−12.7	−9.1	−11.2
Clonitazene	−3.8	−12.8	−16.7	2.6	−20.1	−3.0	−4.6	−15.6
Ethylene etonitazene	8.3	3.8	−2.6	16.7	−4.6	10.8	−4.8	−6.5
Etodesnitazene	−1.5	−6.7	−8.0	6.6	−15.5	3.2	−12.5	−19.3
Etonitazene	−12.1	−11.4	10.1	2.5	−0.1	10.2	−15.9	−11.9
Fluetonitazene	4.6	3.7	17.8	13.3	14.1	18.9	−7.5	−11.6
Flunitazene	23.1	4.8	−3.6	10.3	0.4	−3.7	−6.3	−10.0
*Iso*‐butonitazene	−3.1	−16.4	−14.8	0.9	−23.2	−6.9	−15.9	−20.2
Isotodesnitazene	−25.2	−22.9	−1.0	−2.9	−9.8	6.4	−11.3	−13.3
Menitazene	−14.9	−14.9	10.0	2.5	−3.2	4.9	−9.1	−3.1
Methionitazene	−1.2	8.8	−1.8	9.7	−11.4	5.8	15.9	−6.5
Methylenedioxynitazene	24.4	2.8	−4.2	9.0	0.4	−3.8	1.6	−10.7
Metodesnitazene	3.5	1.7	0.3	9.6	−6.3	12.6	0.0	−11.0
Metonitazene	28.1	12.1	−6.0	8.3	0.6	−6.1	−1.7	−11.2
*N*‐desethyl etonitazene	5.8	1.2	−1.2	12.7	−6.2	12.9	10.1	−5.5
*N*‐desethyl metonitazene	17.8	8.8	6.1	13.7	−8.8	10.9	27.6	0.5
*N*‐desethyl protonitazene	27.9	16.1	4.0	15.9	6.5	1.1	−1.6	−15.9
Nitazene	28.1	5.8	−3.4	12.5	1.9	−1.4	3.3	−6.4
Nitazene dihydrofuran variant	8.1	4.5	24.3	16.1	15.8	17.5	−7.6	−14.4
*N*‐piperidinyl 4′‐hydroxy nitazene	10.5	5.2	10.4	17.4	1.6	25.3	9.2	−0.7
*N*‐piperidinyl etonitazene	4.8	2.0	−8.3	11.7	−8.6	6.6	10.0	−5.1
*N*‐pyrrolidino 4′‐hydroxy nitazene	23.7	−1.1	−1.1	4.2	−0.5	−2.5	−2.5	−5.7
*N*‐pyrrolidino etodesnitazene	25.9	0.5	−11.5	5.0	−5.3	−6.6	0.0	−6.1
*N*‐pyrrolidino etonitazene	24.0	3.4	−8.6	12.7	−0.1	−0.8	3.3	−5.2
*N*‐pyrrolidino isotonitazene	12.8	5.2	1.5	14.6	−6.4	8.7	11.9	0.4
*N*‐pyrrolidino metodesnitazene	24.0	16.4	−7.3	11.1	5.4	−0.2	−1.6	−6.0
*N*‐pyrrolidino metonitazene	−15.7	−9.2	5.2	6.0	6.6	8.8	−8.7	−9.4
*N*‐pyrrolidino protonitazene	−8.9	−8.2	19.5	8.5	5.7	5.6	−10.3	2.8
Propylnitazene	0.1	−16.2	−15.6	3.4	−21.8	−4.8	−11.3	−20.7
Protodesnitazene	22.5	−4.6	−6.5	6.8	−10.5	−8.1	−6.1	−10.1
Protonitazene	−11.0	−17.8	1.8	5.3	−4.7	−5.1	−7.3	7.6
Pyrrolidino variant etonitazene	29.0	−3.1	−1.3	13.8	−1.2	−4.0	−1.6	−2.9
*Sec*‐butonitazene	−17.5	−15.4	0.6	−2.7	−6.7	8.5	−8.7	6.3
Trifluorometonitazene	−20.2	−21.3	22.3	−7.0	−11.4	10.2	6.1	20.2

Freeze–thaw stability, evaluated after 1, 3, and 5 cycles, was more heterogeneous. Deviations after storage were compound‐ and concentration‐dependent, rather than uniformly progressive with increasing cycle number. Deviations were already observed for some compounds, including 4′‐hydroxy nitazene at LQC (+25.9%), alpha‐methyl etonitazene at LQC (+32.4%), and metonitazene at LQC (+28.1%) after the first cycle. Persistent deviations after later cycles were also observed for 5‐aminoisotonitazene at HQC (−33.9% after three cycles and −30.8% after five cycles) and nitazene dihydrofuran variant at LQC (+24.3% after five cycles). These results indicate that freeze–thaw stability was not uniform and should be interpreted in an analyte‐specific manner. This is consistent with the available nitazene literature, which indicates that stability may vary according to the analyte, matrix, concentration, and storage conditions [7]. Therefore, the present results have practical relevance, as nitazene‐positive samples, particularly those intended for quantitative interpretation, should ideally be analyzed promptly and should not undergo unnecessary repeated freeze–thaw cycles.

Previous studies have reported heterogeneous stability profiles for nitazenes across different matrices and storage conditions. In whole blood, Walton et al. [14] reported that room‐temperature stability differed across analogs and that freezer stability was inconsistent for several parent nitazenes, with marked degradation after 28 days of frozen storage. The same study also showed that some compounds remained stable for up to 60 days under selected storage conditions, whereas others showed earlier loss, reinforcing that stability is not uniform across the class. In DBS, Vitrano et al. [6] showed that different nitazenes displayed distinct short‐term and medium‐term stability profiles, depending on concentration and temperature. At trace concentrations (1 ng/mL), some compounds such as isotonitazene and etodesnitazene became undetectable after 30 days of storage, whereas higher concentrations (5 ng/mL) exhibited greater stability. They concluded that no single storage condition was optimal for all compounds. By contrast, Ververi et al. [8] reported no significant variation for nine nitazenes in DBS over 28 days, with room temperature showing the lowest variability. These findings suggest that stability may differ substantially between liquid biological matrices and dried microsamples. In addition, Parks et al. [22] documented the degradation of metonitazene in unpreserved postmortem blood, whereas preserved fluoride/oxalate blood did not show the same behavior. These findings indicate that at least some nitro‐nitazenes may be particularly susceptible to degradation when appropriate preservatives are not used. Accordingly, the present results support a careful analyte‐specific interpretation of nitazene concentrations, especially when sample storage, repeated freezing, or reanalysis is required.

The present study focused on stability conditions relevant to routine handling and short‐term processing, whereas longer‐term stability has only been explored in a limited number of previous studies. The available data, generally covering periods of up to 1–2 months, already suggest that degradation may be variable and difficult to predict, particularly in the context of retrospective casework [6].

#### 3.2.5 Application to an Authentic Intoxication Case

The validated method was successfully applied to an authentic clinical specimen obtained from an intoxication case assisted at Campinas Poison Control Center. Toxicological analysis confirmed the presence of *N*‐pyrrolidino protonitazene at a concentration of 1.4 ng/mL (see Figure S2). Clinically, the case was characterized by marked somnolence, miosis, apnea, oxygen saturation of 30%, and a Glasgow Coma Scale score of 3, indicating severe opioid toxicity with profound central nervous system depression. A total naloxone dose of 1.2 mg, administered as three 1‐mL ampoules containing 0.4 mg/mL each, was required to reverse respiratory failure. Toxicological screening also identified MDA, dipentylone, pentylone, ethanol, and nicotine, indicating polydrug exposure. In addition, qualitative analysis of the corresponding urine sample detected *N*‐pyrrolidino 4′‐hydroxy nitazene, a dealkylated nitazene‐related metabolite, providing further support for nitazene exposure in this case. Although the clinical findings are consistent with severe opioid toxicity and with the pharmacological profile reported for *N*‐pyrrolidino protonitazene, the clinical presentation, including the Glasgow Coma Scale score of 3, should not be attributed solely to this compound. The concomitant detection of other psychoactive substances indicates substantial polydrug exposure and possible additive or synergistic toxic effects [23, 24].

From an analytical perspective, this case demonstrates the applicability of the proposed method to a real toxicological specimen containing a highly potent emerging nitazene at concentration relevant to clinical toxicology. The measured concentration is also plausible considering the published literature, although direct comparison should be interpreted cautiously because most reported values refer to postmortem blood rather than clinical samples. In a forensic case series from North America and the United Kingdom, *N*‐pyrrolidino protonitazene was quantified in blood over a range of 0.3–55 ng/mL, with a median of 1.2 ng/mL [1], while a recent fatal case report described 3.8 ng/mL in postmortem serum, 1.7 ng/mL in heart blood, and approximately 0.52 ng/mL in femoral blood [23]. In addition, previously summarized non‐fatal cases reported whole‐blood concentrations ranging from 0.7 to 3.0 ng/mL, with a mean of 2.2 ng/mL [18]. Accordingly, the concentration observed here falls within the range already described for this analogue and supports the suitability of the method for low‐level detection in clinically relevant cases.

The clinical course observed is consistent with current knowledge on nitazene toxicity. Recent clinical data indicate that nitazene poisoning commonly presents with sedation and hypoventilation, and that repeat naloxone dosing or even continuous infusions are frequently required in severe cases [2, 4, 17]. At the same time, interpretation of concentration–effect relationships remains difficult, particularly in the presence of co‐exposures. Reviews of nitazene‐related cases have emphasized that many published intoxications are multidrug cases, which limits direct conclusions regarding toxic thresholds for individual analogs [15, 18]. Therefore, although the present case cannot establish a concentration associated with isolated *N*‐pyrrolidino protonitazene toxicity, it provides clinically relevant evidence that a concentration of 1.4 ng/mL may be associated with a severe opioid toxidrome requiring repeated naloxone administration in a real‐world setting.

This finding is also relevant from both an epidemiological and toxicological perspective. According to Gonçalves de Araújo et al. [25], the arrival of nitazenes in Brazil is a recent phenomenon, primarily detected by law enforcement in herbal fragments intended for smoking, often mixed with synthetic cannabinoids. However, a clinical case involving *N*‐pyrrolidino protonitazene has not been previously reported in Brazil. This case therefore adds regional and practical relevance to the analytical findings. To our knowledge, it is among the first clinically documented cases in Brazil with analytical quantification of *N*‐pyrrolidino protonitazene in plasma, and the findings contributed to communication with the National Early Warning System, supporting national surveillance and control (scheduling) measures. The successful application of the method to an authentic intoxication case reinforces the value of maintaining sensitive, targeted workflows for emerging nitazenes in both clinical and forensic toxicology. In a field where quantitative clinical data remain limited for many nitazenes, each well‐characterized case contributes to the interpretative framework needed for future toxicological evaluation.

A limited number of LC–MS‐based methods for the analysis of nitazene analogs in biological specimens have been described in the literature (Table S1) [6, 7, 8, 9, 11, 14, 16, 19, 21, 22, 23, 26, 27, 28, 29]. Blood‐based matrices are particularly relevant because of their direct relationship with the pharmacological and toxicological effects of these substances. However, many methods developed for authentic casework are restricted in the number of nitazenes included or require large sample volumes. In this context, the present study describes a method for the simultaneous quantification of a broad nitazene panel in plasma using a miniaturized micro‐QuEChERS approach coupled to LC–MS/MS.

Most methods developed for the determination of these emerging opioids in whole blood or urine rely on conventional sample preparation procedures, particularly LLE. As detailed in Table S1, these traditional procedures often require relatively large volumes of organic solvents, frequently ranging from 1 to 3 mL per sample [11, 14], which may increase cost, sample handling, and solvent consumption. The present method reduces the extraction volume to 400 μL of acetonitrile, offering a more streamlined alternative with lower solvent consumption.

Another relevant aspect is the sample volume required. The developed method used only 100 μL of plasma, while several other studies used larger volumes of biological matrices. For example, Wachełko et al., Walton et al., and Mueller et al. [11, 14, 26] each used 500 μL of whole blood to detect nitazenes using LLE or SPE. While some recent studies used smaller volumes, such as Schüller et al. [7] using 120 μL for LPME or Pardi et al. [21] using 50 μL for protein precipitation, these approaches either required more specialized extraction formats or focused on a more restricted analytical scope.

Despite the reduction in both sample and solvent volumes, the present micro‐QuEChERS method showed suitable analytical sensitivity, with an LLOQ of 0.05 ng/mL and an LOD of 0.025 ng/mL for the validated analytes. While assays utilizing larger sample volumes (500 μL) have reported LLOQs ranging from 0.01 to 0.5 ng/mL, previously published methods working with 100 μL or less typically reported higher quantification limits, such as 1.0 ng/mL. Overall, the validated approach provides a practical alternative for nitazene quantification, combining broad analytical scope, reduced sample volume, operational simplicity, and sensitivity compatible with routine application in forensic and clinical toxicology laboratories.

## 4 Conclusions

A targeted LC–MS/MS method was successfully developed and validated for the quantification of nitazene analogs in plasma using micro‐QuEChERS sample preparation. The method showed suitable analytical performance for the final validated panel and proved applicable to an authentic intoxication case, supporting its use in real toxicological settings. Given the increasing global emergence of nitazenes and their growing clinical and forensic relevance, this workflow provides a practical and sensitive approach for targeted analysis, strengthening analytical capacity in clinical and forensic toxicology.

## Author Contributions

**Karla A. O. Souza:** methodology, validation, formal analysis, investigation, writing – original draft, writing – review and editing, visualization. **Náthaly C. B. F. Santos:** methodology, investigation, writing – original draft, writing – review and editing. **Rafael Lanaro:** methodology, investigation, writing – original draft. **Sara E. Walton:** writing – review and editing, supervision. **Alex J. Krotulski:** writing – review and editing, supervision. **Barry K. Logan:** writing – review and editing, supervision. **Mauricio Yonamine:** writing – review and editing, supervision. **Jose Luiz Costa:** conceptualization, resources, writing – original draft, writing – review and editing, supervision, project administration, funding acquisition.

## Funding

This work was supported by Fundação de Amparo à Pesquisa do Estado de São Paulo‐FAPESP (Process Number 2020/10809‐5, 2021/04768‐7, 2023/07323‐1 and 2024/15262‐5), Conselho Nacional de Desenvolvimento Científico e Tecnológico‐CNPq (Process Numbers 315640/2021‐9; 403396/2024‐7; INCT‐SP 406958/2022‐0) and Coordenação de Aperfeiçoamento de Pessoal de Nível Superior (CAPES, Finance code 001, INSPEQT 2.0 Project).

## Ethics Statement

All procedures performed in studies involving human participants were in accordance with the ethical standards of the University of Campinas Committee (Comitê de Ética em Pesquisa da UNICAMP – CEP, CAAE 46404121.8.0000.5404).

## Conflicts of Interest

The authors declare no conflicts of interest.

## Supporting information


**Figure S1:** Representative LC–MS/MS extracted ion chromatograms of all analytes at the LLOQ.
**Figure S2:** Representative LC–MS/MS chromatograms of (a) blank plasma and (b) the authentic intoxication case sample positive to N‐pyrrolidino protonitazene (1.4 ng/mL).
**Table S1:** Analytical methods for detecting nitazenes in different sample types.
**Table S2:** Exogenous substances analyzed at a concentration of 200 ng/mL for potential interference with the method.
**Table S3:** New psychoactive substances analyzed at a concentration of 100 ng/mL for potential interference with the method.

## Data Availability

The data are available from the corresponding author upon reasonable request.
