# Supplementary material for: Micro‐QuEChERS Extraction for Broad‐Spectrum LC‐QQQ‐MS Quantification of Nitazene Analogs in Plasma: A Validated Method With Casework Application
Source: Drug Test Anal. 2026 Jul 28;18(10):1296–310. doi: 10.1002/dta.70126 (PMC13628430; doi:10.1002/dta.70126)
Supplement: Supplementary file 1 [file DTA-18-1296-s001.docx]

**Supplementary Material**

**Micro-QuEChERS extraction for broad-spectrum LC-QQQ-MS quantification of nitazene analogs in plasma: a validated method with casework application**

**Contents of this file**

1. **Supplementary figures**

Figure S1. Representative LC-MS/MS extracted ion chromatograms of all analytes at the LLOQ.

Figure S2. Representative LC-MS/MS chromatograms of (a) blank plasma and (b) the authentic intoxication case sample positive to *N*-pyrrolidino protonitazene (1.4 ng/mL).

2. **Supplementary tables**

Table S1. Analytical methods for detecting nitazenes in different sample types.

Table S2. Exogenous substances analyzed at a concentration of 200 ng/mL for potential interference with the method.

Table S3. New Psychoactive Substances analyzed at a concentration of 100 ng/mL for potential interference with the method.

**Figure S1.** Representative LC-MS/MS extracted ion chromatograms of all analytes at the LLOQ.

**Figure S2.** Representative LC-MS/MS chromatograms of (a) blank plasma and (b) the authentic intoxication case sample positive to *N*-pyrrolidino protonitazene (1.4 ng/mL).

**Table S1.** Analytical methods for detecting nitazenes in different sample types.

| Analytes | Biological specimen | Extraction procedure | Instrument | Stationary phase | LOQ | Calibration range | Reference |
| --- | --- | --- | --- | --- | --- | --- | --- |
| Etazene, nitazene, 5-methyl-etodesnitazene, isotodesnitazene, protodesnitazene, flunitazene, metonitazepyne, metonitazene, clonitazene, etonitazepyne, metonitazepipne, ethyleneoxynitazene, etonitazene, etonitazepipne, isotonitazene, protonitazene, isotonitazepipne, protonitazepipne, butonitazene, *iso*-butonitazene, *sec*-butonitazene, 4'-OH-nitazene, *N*-pyrrolidino-4'-OH-nitazene, N-desethyl-etonitazene, *N*-desethyl-isotonitazene, 5-amino-isotonitazene. | Blood 500 μL | LLE 2 mL of ethyl acetate | UHPLC-MS/MS | ReproShell PFP (2.7 µm) and Acquity BEH C18 (1.7 µm) | 0.01 ng/mL | 0.01-15 ng/mL | ^11^ |
| Clonitazene, etodesnitazene, etonitazene, etonitazepyne, flunitazene, isotonitazene, metodesnitazene, metonitazene, protonitazene. | Blood  120 µL | LPME (4 µL organic membrane, 50 µL aqueous acceptor) | UHPLC-MS/MS | Kinetex Biphenyl (100 × 2.1 mm, 1.7 µm) | 0.1–0.5 nM | 0.1–50 nM | ^7^ |
| Isotonitazene, metonitazene, protonitazene, etonitazene, clonitazene, flunitazene, *N*-desethyl isotonitazene, 5-amino isotonitazene, 4′-hydroxy nitazene. | Blood  Urine  500 µL | LLE  3 mL *N*-butyl chloride:ethyl acetate | LC-MS/MS | Agilent Poroshell 120 C18 (100 × 3.0 mm, 2.7 µm) | 0.5 ng/mL | 0.5–50 ng/mL | ^14^ |
| Isotonitazene, metonitazene. | Plasma, blood, urine, brain, liver 50 µL | PPT  150 µL acetonitrile or ethyl acetate | LC-MS/MS | Agilent Poroshell 120 EC-C18 (100 × 2.1 mm, 2.7 µm) | 0.10 ng/mL (blood / plasma) and 1.0 ng/mL (urine / liver) | 0.1–100 ng/mL | ^21^ |
| Isotonitazene | blood, urine, fluids (500 µL), tissues (1 g) | SPE 2 mL DCM:MeOH:NH3 elution | UHPLC-MS/MS | Accucore RP-MS C18 (50 × 2.1 mm, 2.6 µm) | 0.05 ng/mL (blood) | 0.05–10 ng/mL | ^26^ |
| *N*-pyrrolidino protonitazene | Serum, plasma, urine (100 µL) | PPT  1 mL acetonitrile | LC-MS/MS (QTRAP) | Phenomenex Kinetex F5 (100 × 2.1 mm, 2.6 μm) | 1.0 ng/mL | 1.0–20 ng/mL | ^23^ |
| *N*-pyrrolidino etonitazene, butonitazene, etodesnitazene, etonitazepipne, flunitazene, isotonitazene, metodesnitazene, metonitazene, protonitazene, brorphine. | Dried blood spots / DBS (10 µL) | Solvent extraction (500 µL MeOH:ACN) and ultrasonication | UHPLC-MS/MS | Phenomenex Kinetex C18 (100 × 2.1 mm, 1.7 µm) | 1.0 ng/mL | 1.0–50 ng/mL | ^8^ |
| Etazene, flunitazene, isotonitazene, protonitazene. | Dried blood spots / DBS (30 µL) | Solvent extraction (500 µL methanol) and ultrasonication | LC-HRMS/MS (Orbitrap) | FORCE Biphenyl (50 × 3.0 mm, 3 µm) | 0.5 ng/mL | 1.0–20 ng/mL | ^6^ |
| Metodesnitazene, etodesnitazene, 4'-hydroxy nitazene, *N*-desethyl etonitazene, *N,N*-dimethylamino etonitazene, flunitazene, 5-amino isotonitazene, metonitazene, *N*-desethyl isotonitazene, *N*-pyrrolidino etonitazene, ethyleneoxynitazene, *N*-pyrrolidino protonitazene, *N*-piperidinyl etonitazene, isotonitazene, protonitazene, butonitazene, *sec*-butonitazene. | Hair (20 mg) | Cryogenic grinding + ultrasonic extraction (1 mL Methanol) | UHPLC-MS/MS (QTRAP) | Phenomenex Kinetex Biphenyl (100 × 3.0 mm, 2.6 µm) | 5–20 pg/mg | 5–1000 pg/mg | ^27^ |
| Isotonitazene. | Hair (20 mg) | Solvent extraction (1 mL Methanol) and ultrasonication | LC-MS/MS | Ascentis Express C18 (100 × 2.1 mm, 2.7 μm) | 2.5 pg/mg | 2.5–250 pg/mg | ^28^ |
| 5-amino isotonitazene, 5-methyl etodesnitazene, butonitazene, clonitazene, etodesnitazene, etonitazene, flunitazene, isotonitazene, metodesnitazene, metonitazene, *N*-desethyl isotonitazene, *N*-piperidinyl etonitazene, *N*-pyrrolidino etonitazene, *N*-pyrrolidino metonitazene, *N*-pyrrolidino protonitazene, protonitazene. | Blood, urine | Multi-drug extraction (not specified) | LC-MS/MS (QTRAP) | Phenomenex Kinetex F5 (100 × 2.1 mm, 2.6 μm) | 0.1–0.5 ng/mL | 0.5–80 ng/mL | ^9^ |
| Metonitazene, 5-aminometonitazene, 5-acetamidometonitazene | Blood  200 µL | LLE (2 mL MTBE) | LC-QQQ-MS & LC-QTOF-MS | Agilent Poroshell EC-C18 (100 × 2.1 mm, 2.7 µm) | 0.5 ng/mL | 0.5–100 ng/mL | ^22^ |
| 4′-hydroxy nitazene, 5-methyl etodesnitazene, isotonitazene, metodesnitazene, protonitazene, *N*-piperidinyl etonitazene, *N*-pyrrolidino etonitazene. | Blood | LLE (1-chlorobutane) | LC-MS/MS (Precursor Ion Scan mode) | Agilent Poroshell 120 EC-C18 (100 × 2.1 mm, 2.7 µm) | 0.5 ng/mL (LOD) | N/A (Screening cutoff at 1.0 ng/mL) | ^29^ |
| Metonitazene | Blood Urine (1mL) | enzymatic hydrolysis | LC-MS/MS  LC-QTOF-MS | l–column 2 ODS semi–micro | 0.1 ng/mL and 0.5 ng/mL | - | ^16^ |
| Metonitazene | 500 μL  blood | LLE 3 mL of 1-chlorobutane and ethyl acetate (70:30, v:v | LC-MS/MS  LC-QTOF-MS | Poroshell 120 EC-C18 | 0.1 ng/mL | 0.5–50 ng/mL | ^19^ |
| 4′-hydroxy nitazene, 5-aminoisotonitazene, 5-methyl etodesnitazene, 5-trifluoromethyl isotonitazene, alpha-methyl etonitazene, butonitazene, clonitazene, ethylene etonitazene, etodesnitazene, etonitazene, fluetonitazene, flunitazene, *iso*-butonitazene, isotodesnitazene, menitazene, methionitazene, methylenedioxynitazene, metodesnitazene, metonitazene, *N*-desethyl etonitazene, *N*-desethyl metonitazene, *N*-desethyl protonitazene, nitazene, nitazene dihydrofuran variant, *N*-piperidinyl 4′-hydroxy nitazene, *N*-piperidinyl etonitazene, *N*-pyrrolidino 4′-hydroxy nitazene, *N*-pyrrolidino etodesnitazene, *N*-pyrrolidino etonitazene, *N*-pyrrolidino isotonitazene, *N*-pyrrolidino metodesnitazene, *N*-pyrrolidino metonitazene, *N*-pyrrolidino protonitazene, propylnitazene, protodesnitazene, protonitazene, pyrrolidino variant etonitazene, *sec*-butonitazene, and trifluorometonitazene | Plasma 100 μL | Micro-QuEChERS | LC-MS/MS | Phenomenex Kinetex C18 (3.0 mm × 50 mm, 2.6 μm) | 0.025 ng/mL | 0.05–20 ng/mL | Proposed study |

**Table S2.** Exogenous substances analyzed at a concentration of 200 ng/mL for potential interference with the method.

Pharmaceuticals

Haloperidol, risperidone, quetiapine, hydrochlorothiazide, chlorthalidone, furosemide, atenolol, propranolol, diltiazem, captopril, phenobarbital, phenytoin, carbamazepine, valproic acid, ondansetron, ranitidine, metoclopramide, domperidone, albuterol, naphazoline, glyburide, atorvastatin, desloratadine, loratadine, hydroxyzine, promethazine, acetaminophen, meloxicam, prednisone, prednisolone, celecoxib, betamethasone, dexamethasone, desmethylvenlafaxine, venlafaxine, bupropion, amitriptyline, clomipramine, fluoxetine, nortriptyline, mirtazapine and escitalopram.

**Table S3.** New Psychoactive Substances analyzed at a concentration of 100 ng/mL for potential interference with the method.

New Psychoactive Substances

5F-EMB-PINACA, ADB-BINACA / ADB-BUTINACA, AB-FUBINACA, MMB-FUBINACA, 4F-MDMB-BUTICA, ADB-4en-PINACA, RCS-4, RCS-8, JWH-015, JWH-203, JWH-210, AM-2233, 5F-BZO-POXIZID, 5F-MDMB-PINACA, AB-PINACA, JWH-175, 4F-ABUTINACA, ADAMANTYL-CHMINACA, ADB-BENZINACA/ADB-BINACA, PB-22, APINACA, JWH-250, JWH-200, HU-211, BZO-HEXOXIZID, BZO-POXIZID, 5F-EMB-PICA, 4CN-MMB-BUTINACA, 5F-EDMB-PICA, BZO-CHMOXIZID, ADB-FUBIATA, MDMB-4en-PINACA, 4F-MDMB-BUTINACA, JWH-176, XLR-11, JWH-019, JWH-122, JWH-081, AM-2201, ADB-HEXINACA, JWH-073, amphetamine, methamphetamine, fenproporex, 3,4-methylenedioxymethamphetamine (MDMA), 3,4-methylenedioxyethylamphetamine (MDEA), para-methoxyamphetamine (PMA), 4-methylthioamphetamine (4-MTA), 2-MAPB, 4(5*-6*)MAPB, ritalinic acid, ethylphenidate, trifluoromethylphenylpiperazine (TFMPP), meta-chlorophenylpiperazine (mCPP), LDX, MDDMA, 1-benzylpiperazine (BZP), methylphenidate, cathinone, metcathinone, butylone, pentylone, N-ethylpentylone, mephedrone, bk-MDMA (methylone), N-ethylheptedrone, bk-DMBDP (dipentylone), bk-MDDMA (dimethylone), eutylone, alpha-PiHP, 2-methylmethcathinone (2-MMC), methedrone, pentedrone, benzedrone, 4-chloroethcathinone, flualprazolam, clonazolam, 8-aminoclonazolam, bromazolam, deschloroetizolam, flubromazepam, flubromazolam, N-desalkylflurazepam, etizolam, acetylfentanyl, acetylnorfentanyl, acrylfentanyl, alfentanil, carfentanil, fentanyl, furanylfentanyl, norfentanyl, remifentanil, sufentanil, thiofentanyl, valerylfentanyl, 3-methoxy-PCP, ketamine, norketamine, 2F-deschloroketamine, hydroxyketamine, deschloroketamine, DOET, DOM, mescaline, harmine, harmaline, lysergic acid diethylamide (LSD), methylenedioxypyrovalerone (MDPV), lysergic acid amide (LSA), 5-MeO-MiPT, N,N-dimethyltryptamine (DMT), 2-oxo-3-hydroxy-LSD, psilocin, 2C-C, 2C-D, 2C-B, 2C-T, 2C-G, 2C-I, 25E-NBOH, 25I-NBOMe, 25H-NBOMe, 2C-T-4, 2C-E, 2C-T-2, 25C-NBOH, 25B-NBOH, 25I-NBOH, 25N-NBOMe, 25B-NBOMe, 25C-NBOMe, 25T-NBOMe.
